# Exploratory spatial transcriptomic profiling of peritumoral Th2 immune polarization in HPV-positive oropharyngeal cancer

**DOI:** 10.3389/fonc.2025.1732480

**Published:** 2025-11-26

**Authors:** Naohiro Wakisaka, Makiko Moriyama-Kita, Satoru Kondo, Eiji Kobayashi, Takayoshi Ueno, Yosuke Nakanishi, Kazuhira Endo, Hisashi Sugimoto, Tomokazu Yoshizaki

**Affiliations:** 1 Department of Otorhinolaryngology, NHO Kanazawa Medical Center, Kanazawa, Japan; 2 Division of Otorhinolaryngology and Head and Neck Surgery, Graduate School of Medical Science, Kanazawa University, Kanazawa, Japan

**Keywords:** HPV-positive oropharyngeal cancer, palatine tonsil, spatial transcriptomics, tumor immune microenvironment, Th2 immunity, lymphatic metastasis, eosinophils and mast cells, estrogen signaling

## Abstract

**Introduction:**

Oropharyngeal squamous cell carcinoma (OPC) associated with high-risk human papillomavirus (HPV), particularly HPV-16, generally shows favorable outcomes yet paradoxically exhibits a high incidence of early lymphatic metastasis. The immune mechanisms underlying this phenomenon remain unclear.

**Methods:**

We conducted an exploratory spatial transcriptomic analysis using the GeoMx Digital Spatial Profiler on formalin-fixed, paraffin-embedded samples from six patients with palatine tonsil-derived OPC. Tumor tissue regions (TTRs) and lymphoid follicular regions (LFRs) were compared according to HPV status and nodal involvement.

**Results:**

In HPV-positive LFRs, pathways related to B-cell apoptosis appeared downregulated, suggesting prolonged B-cell survival and antigen presentation. Metastasis-negative HPV-positive cases displayed a Th2-skewed immune profile in LFRs, with increased naïve B cells, plasma cells, eosinophils, M2 macrophages, and activated mast cells. In contrast, metastasis-positive cases showed increased T cell activation in LFRs and reduced proliferation-related signaling in TTRs. Pathways involving estrogen signaling and bile acid metabolism were also associated with metastatic behavior.

**Conclusion:**

These exploratory findings suggest that peritumoral Th2-biased immunity within lymphoid structures may contribute to restraining lymphatic metastasis in HPV-positive OPC. Spatial transcriptomics may provide a high-resolution framework for investigating tumor–immune interactions and generating hypotheses for future mechanistic and clinical studies.

## Introduction

Oropharyngeal squamous cell carcinoma (OPC), a subtype of head and neck squamous cell carcinoma, is increasingly driven by high-risk human papillomavirus (HPV), particularly HPV-16, rather than traditional risk factors such as tobacco and alcohol. The 8th edition of the Union for International Cancer Control (UICC) staging system (1) now classifies HPV-positive and HPV-negative OPC as distinct entities, reflecting their different molecular features and clinical behaviors (2, 3). Although HPV-positive OPC exhibits a rapidly increasing incidence and generally favorable prognosis, it paradoxically shows a high frequency of early lymphatic metastasis, suggesting distinct virus-driven tumor–immune interactions that remain poorly understood (3).

Immune responses in HPV-positive OPC have been mainly characterized in terms of T-cell activity, as HPV-specific lymphocytes are frequently detected within tumors (4). However, recent studies indicate that B-cell–rich lymphoid structures and Th2-polarized immune microenvironments may modulate the balance between antitumor and tumor-promoting immunity (5–7). Th2 polarization in the tumor microenvironment has been implicated in tumor-promoting processes mediated by IL-4 and IL-13 signaling, which enhance immune evasion, tissue remodeling, and lymphangiogenesis through M2-like macrophage activation (8, 9). Through antigen presentation and crosstalk with T helper subsets, B cells may represent key modulators of peritumoral lymphoid immunity.

Our previous transcriptomic analysis of peritumoral tonsillar tissues suggested that adjacent lymphoid regions act as immune hubs influencing nodal metastasis (10). However, bulk RNA profiling lacks spatial resolution to localize specific immune niches or cellular interactions.

Spatial transcriptomics enables *in situ* mapping of gene expression within defined tissue compartments. Using the GeoMx Digital Spatial Profiler (DSP), immune and transcriptional heterogeneity can be examined directly within tumor tissue regions (TTRs) and lymphoid follicular regions (LFRs) (11). In line with this approach, recent spatial transcriptomic studies in head and neck squamous cell carcinoma have begun to elucidate spatial immune heterogeneity and its association with HPV status and therapeutic response (12–14).

In this exploratory study, we applied spatial transcriptomic profiling to HPV-positive OPC to delineate immune features within peritumoral lymphoid structures and to explore how Th2 polarization and B-cell–associated immunity might influence lymphatic metastasis.

## Materials and methods

### Study design

In this exploratory study, spatial transcriptomic profiling was performed on formalin-fixed, paraffin-embedded (FFPE) tissues obtained from radical resections of palatine tonsil-derived OPC (15). Regions of interest (ROIs) representing tumor tissue regions (TTRs) and lymphoid follicular regions (LFRs) were analyzed using the GeoMx DSP platform to explore immune features associated with HPV status and lymph node metastasis.

### Patient cohort

Six FFPE samples from primary tonsillar OPCs resected at Kanazawa University Hospital (2019–2020) were analyzed. All patients were male; four were HPV-positive and two HPV-negative, including three metastasis-negative and three metastasis-positive cases. Staging followed the 8th edition of the TNM classification (1). HPV status was determined by p16 immunohistochemistry and confirmed by HPV DNA PCR using type-specific primers for high-risk HPV genotypes. Cases showing diffuse (≥70%) nuclear and cytoplasmic p16 staining were classified as HPV-positive. No patient received adjuvant radiotherapy or chemotherapy. Clinical details are summarized in **Supplementary Table S1**. Although the cohort was small, age and stage distributions were comparable between HPV-positive and HPV-negative groups. No formal adjustment for clinicopathologic variables was performed due to the exploratory nature of the study.

This study was approved by the Kanazawa University Hospital Institutional Review Board (approval number 2016–033) and conducted in accordance with the Declaration of Helsinki. Written informed consent was obtained from all participants. Some samples were previously used in other studies, but the present analysis focused on newly generated spatial transcriptomic data addressing distinct immune features.

### GeoMx whole-transcriptome analysis

**Figure 1:**
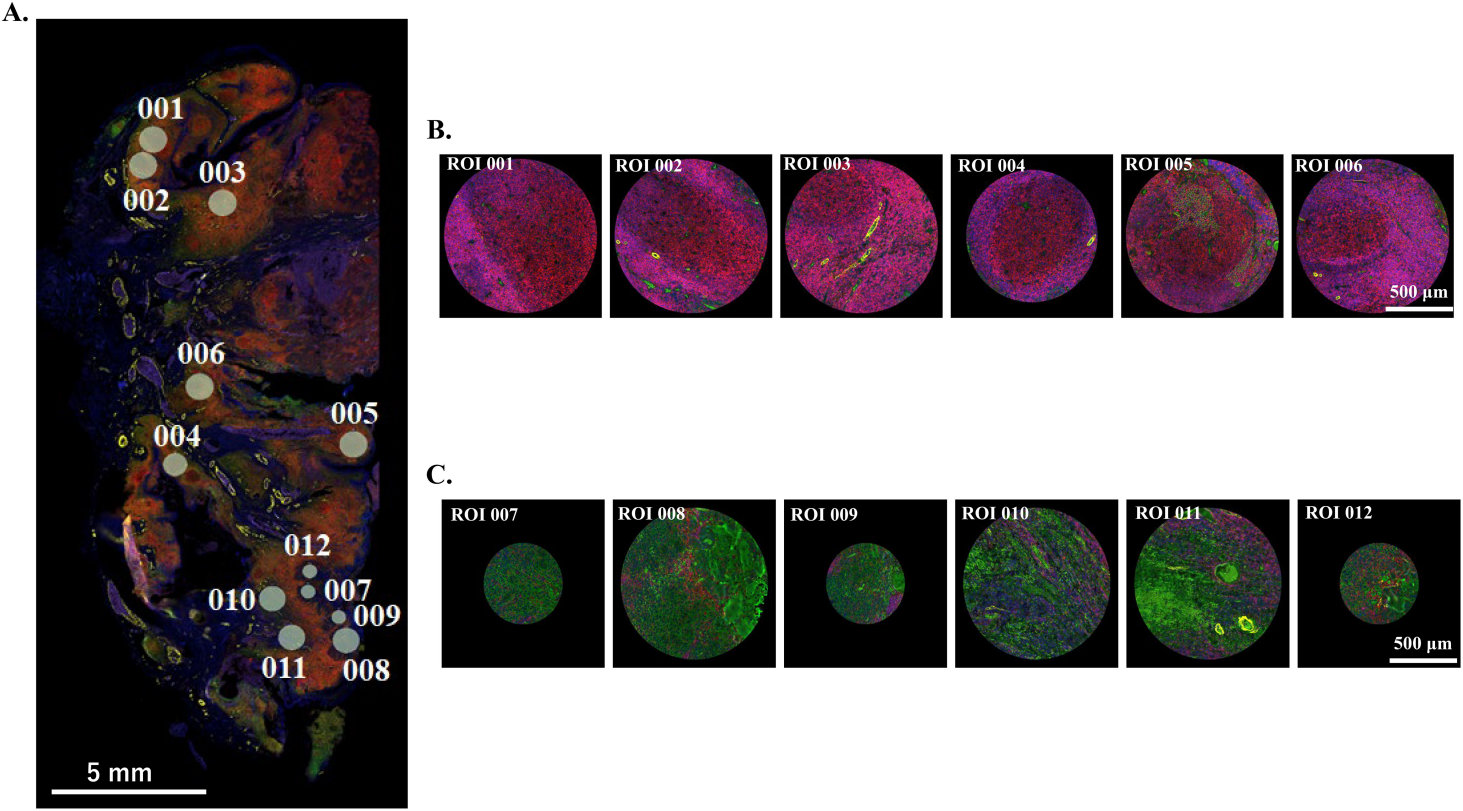
Representative images of a resected HPV-positive oropharyngeal tumor specimen used for GeoMx whole transcriptome atlas analysis. **(A)** Low-power fluorescence microscopy image of a formalin-fixed, paraffin-embedded oropharyngeal tumor section stained for PanCK (green, epithelial/tumor cells), CD45 (red, immune cells), SMA (yellow, stromal cells), and DNA (blue). **(B)** High-power views of selected lymphoid follicular regions (LFRs) (ROIs 001–006), showing well-organized immune cell aggregates corresponding to lymphoid follicular structures. C: High-power views of selected tumor tissue regions (TTRs) (ROIs 007–012), highlighting tumor epithelial components and adjacent stromal regions. White scale bars indicate 5 mm **(A)** and 500 μm **(B, C)**.

Spatial transcriptomic profiling was performed using the GeoMx DSP (NanoString Technologies, Seattle, WA), which enables high-plex RNA detection in FFPE samples (11, 16). The GeoMx Whole Transcriptome Atlas (WTA) interrogates ~18,000 protein-coding genes curated by the Human Gene Nomenclature Committee and RefSeq databases. Sections (5 μm) were mounted on Superfrost Plus slides (VWR, Radnor, PA). Morphology markers (PanCK, CD45, SMA) were used to distinguish epithelial, immune, and stromal compartments. **Figure 1** shows representative fluorescent staining and ROI selection.

Twelve ROIs were selected per specimen (six LFRs and six TTRs), yielding 36 of each across all six patients. ROI selection was performed independently by two independent researchers (M.M.-K. and N.W.) blinded to HPV and nodal status. Data were processed with the DSP Data Analysis Suite using Q3 normalization. A linear mixed model was applied to detect differentially expressed genes (DEGs) between groups. Statistical significance was defined as |log_2_| of fold-change > 0.5 and -log_10_ P > 1.3. Differential expression was assessed using a threshold of unadjusted p < 0.05, which was considered appropriate for this hypothesis-generating, small-cohort spatial transcriptomics study. FDR correction did not yield significant hits owing to small sample size, but consistent trends were validated across independent analyses (ssGSEA, immunohistochemistry).

### Pathway and network analysis

Functional annotation of DEGs was performed using the ClueGO plugin for Cytoscape (v3.10.3; https://cytoscape.org) to identify enriched Gene Ontology (GO) categories including Biological Process, Cellular Component, and Immune System Process (17).

### Immune cell deconvolution

Relative immune cell proportions were estimated from ROI expression profiles using CIBERSORTx (https://cibersortx.stanford.edu/) based on 22 immune subsets (18). Outputs were interpreted as relative rather than absolute, given the lack of OPC-specific single-cell reference datasets.

### Single-sample gene set enrichment analysis

Gene set enrichment was evaluated by single-sample GSEA (ssGSEA) using hallmark gene sets from the Molecular Signatures Database (MSigDB; https://www.gsea-msigdb.org/gsea/index.jsp). Analyses were performed using GenePattern software v3.9.11 and the ssGSEA module v10.1.0 (19).

### Immunohistochemistry

Consecutive 5 μm sections from FFPE blocks were immunostained as described previously (20, 21). Mouse monoclonal antibodies against eosinophil major basic protein (EMBP; 1:20) and mast cell tryptase (1:10,000; Bio-Rad, Hercules, CA) were used. Visualization was achieved with diaminobenzidine and methyl green counterstaining. Eosinophilic nasal polyps and inflamed tonsils served as positive controls; nonimmune serum was used for negative controls. For each specimen, three high-power fields (200×) were independently reviewed by two blinded observers with >90% concordance. The staining was evaluated qualitatively to confirm spatial expression patterns and cellular localization; no quantitative scoring (e.g., H-score or positive cell ratio) was applied due to the exploratory nature of the study.

### Statistical analysis

Normalized expression data were used for differential analyses. Linear mixed modeling incorporated patient ID as a random effect, with HPV status and tissue region as fixed factors. Principal component analysis (PCA) was applied to normalized LFR profiles to visualize clustering and exclude batch effects. The Mann–Whitney U test was used for two-group comparisons. Correlations between enrichment scores and immune cell proportions were assessed by Spearman’s rank test.

For exploratory DEG analyses using GeoMx whole-transcriptome data, statistical significance was defined as unadjusted p < 0.05 (−log10 p > 1.3) and |log2 fold change| > 0.5. Given the exploratory nature of this analysis, these thresholds were used to identify potential immune-related transcriptional patterns. For subsequent validation analyses, including enrichment score correlations and immune cell density comparisons, statistical significance was defined as p < 0.01 to minimize false-positive findings. Given the small cohort size and exploratory nature of the study, no formal power calculation was performed.

## Results

### Overview of spatial transcriptomic profiles

PCA of normalized gene expression from LFRs confirmed the absence of batch effects (**Supplementary Figure S1**). Within HPV-positive cases, modest separation by metastatic status (F = 0.221, p = 0.028) suggested biologically meaningful heterogeneity rather than technical bias. PCA of TTRs revealed diffuse clustering, consistent with the inherent cellular heterogeneity of tumor compartments.

### Distinct transcriptional landscapes by HPV status

Spatial transcriptomic profiles differed markedly between HPV-positive and HPV-negative OPCs (**Supplementary Figure S2**).

In TTRs, 163 differentially expressed genes (DEGs) were detected, with HPV-negative tumors showing upregulation of pathways related to cellular transport and differentiation, while HPV-positive tumors showed no enriched GO terms. This lack of enrichment likely reflects relatively uniform transcriptional profiles among HPV-positive tumors, suggesting lower pathway-level variability or more homogeneous immune activation patterns, rather than technical limitations.

In LFRs, HPV-negative cases demonstrated activation of transcriptional programs related to RNA processing and nucleosome formation, whereas HPV-positive LFRs exhibited enrichment only in the “negative regulation of B cell apoptotic process,” suggesting enhanced B cell survival.

These findings support distinct immune and transcriptional landscapes between HPV-positive and -negative OPCs, consistent with their classification as biologically separate diseases (1–3).

### Transcriptional differences by nodal metastasis in HPV-positive OPCs

Analyses were next restricted to HPV-positive cases (n = 4) to explore lymphatic dissemination. Given the limited sample size and exploratory aim of this study, differential expression was assessed using an unadjusted p < 0.05 threshold, as FDR correction was overly conservative and yielded no significant hits.

In TTRs, metastasis-positive tumors upregulated genes associated with necrotic cell death and chromatin regulation, while metastasis-negative cases showed enrichment of immune activation and keratin-related pathways (**Supplementary Figures S3A–C**).

In LFRs, metastasis-positive cases upregulated pathways linked to T cell activation and antigen processing, whereas metastasis-negative cases showed enrichment of cell proliferation and receptor regulation terms (**Supplementary Figures S3D–F**).

**Figure 2:**
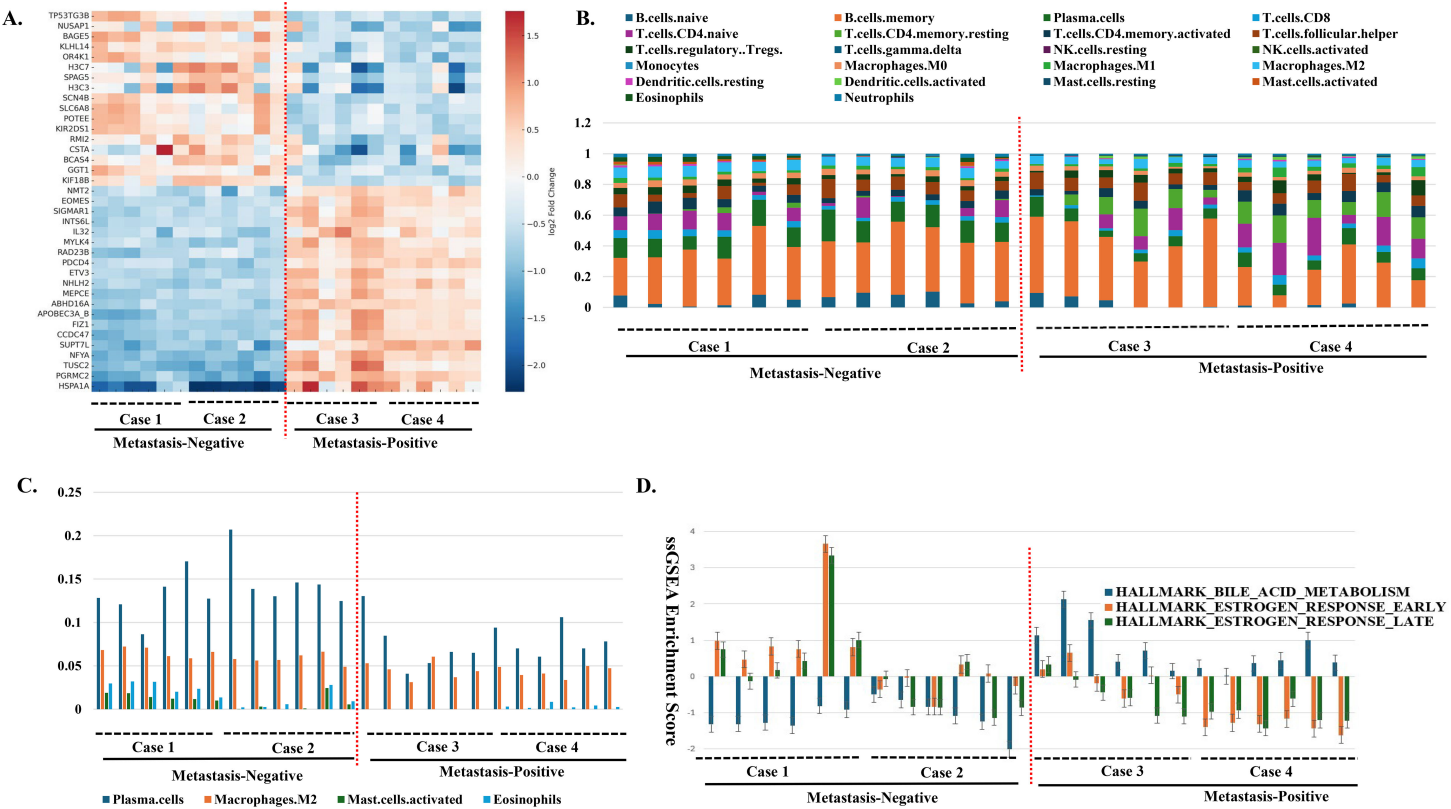
Spatial representation of key transcriptomic features in human papillomavirus (HPV)-positive OPC lymphoid-rich regions (LFRs), analyzed within defined regions of interest (ROIs). A red dashed line in panels **(A–D)** indicates the division between metastasis-negative (left) and metastasis-positive (right) ROIs. **(A)** Heatmap of 40 representative differentially expressed genes, shown as log_2_ fold change relative to the gene-wise mean (color scale as indicated). The upper 20 genes are upregulated in metastasis-negative ROIs, whereas the lower 20 are upregulated in metastasis-positive ROIs. **(B)** Immune cell composition estimated by CIBERSORTx deconvolution of spatial transcriptomic data from these ROIs, showing the relative proportions of immune subsets for each ROI. **(C)** Immune cell composition estimated by CIBERSORTx deconvolution of spatial transcriptomic data from these ROIs, highlighting the relative proportions of plasma cells, M2 macrophages, activated mast cells, and eosinophils—cell populations typically associated with Th2-polarized immune responses (see also **Supplementary Figure S4**). **(D)** ssGSEA enrichment scores for selected immune and signaling pathways in the same LFR ROIs, highlighting bile acid metabolism and estrogen response pathways (see also **Supplementary Figure S5**).

Spatial heatmap of selected DEGs in LFRs, illustrating distinct transcriptional patterns between non-metastatic and metastatic HPV-positive OPCs (**Figure 2A**).

### Immune cell deconvolution in HPV-positive OPCs

Immune cell composition estimated by CIBERSORTx revealed a Th2-skewed phenotype in non-metastatic tumors (**Figures 2B, C**; **Supplementary Figure S4**).

In TTRs of metastasis-negative cases, naïve B cells, M2 macrophages, monocytes, activated mast cells, and eosinophils were significantly enriched, suggesting a Th2-skewed profile.

In LFRs, plasma cells, naïve B cells, M2 macrophages, activated mast cells, eosinophils, and neutrophils were enriched. In contrast, metastasis-positive cases showed enrichment of activated dendritic cells in LFRs, indicating a shift toward Th1 responses.

### Gene set enrichment and immune correlations

ssGSEA analysis using hallmark gene sets revealed gene set enrichment differences based on nodal status (**Figures 2D**: **Supplementary Figure S5**).

In LFRs, inflammatory and interferon response signatures predominated in metastasis-positive cases, while “HALLMARK_ESTROGEN_RESPONSE_EARLY” and “LATE” were enriched in metastasis-negative cases. In contrast, these estrogen-response enrichments were not detected in the TTRs, indicating that hormone-responsive signaling was compartment-specific to the peritumoral LFRs. Conversely, bile-acid-metabolism pathways were upregulated in LFRs of metastasis-positive cases but absent in TTRs, suggesting a metabolic activation associated with lymphatic dissemination within the peritumoral compartment.

**Figure 3:**
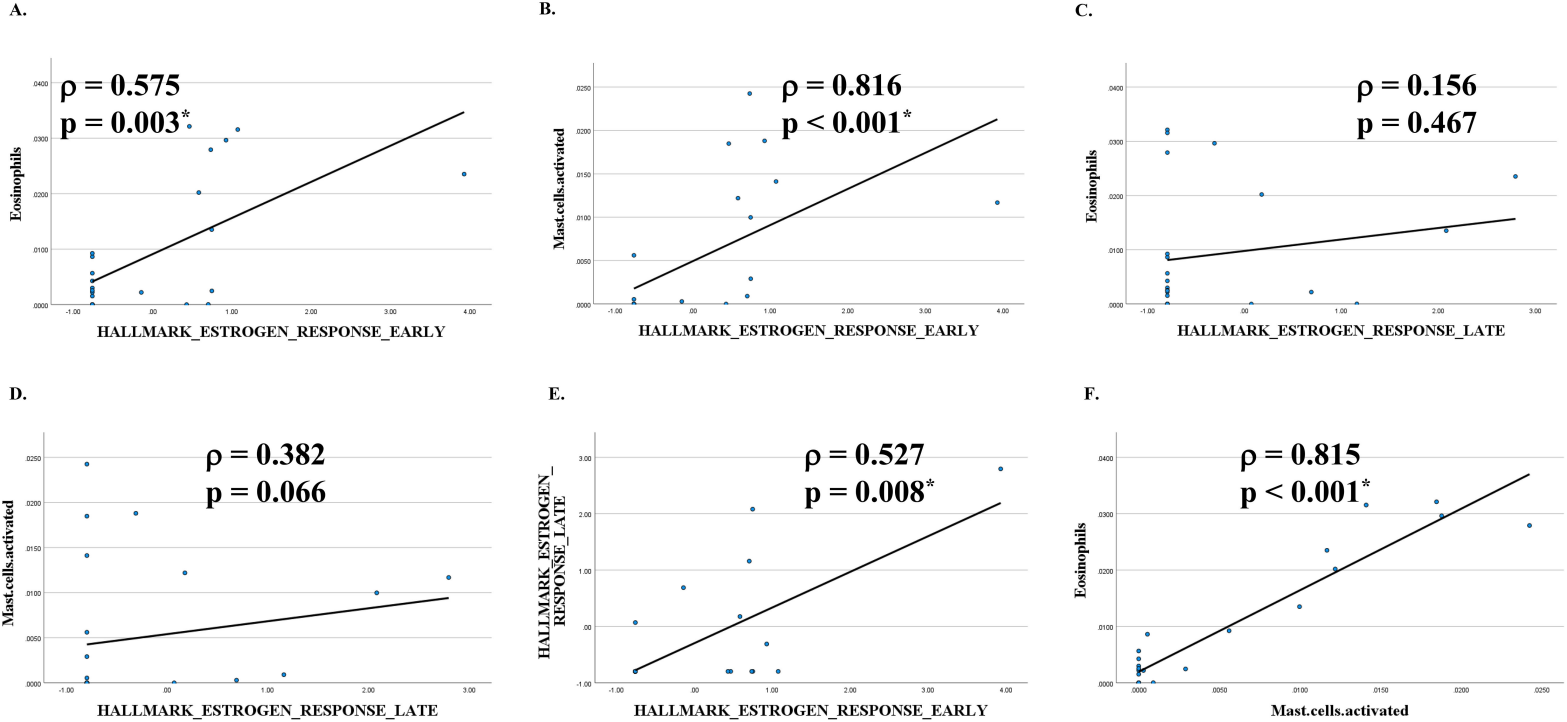
Correlations between estrogen response enrichment scores and immune cell proportions in HPV-positive oropharyngeal carcinoma. Enrichment scores (ESs) for HALLMARK_ESTROGEN_RESPONSE_EARLY and HALLMARK_ESTROGEN_RESPONSE_LATE were calculated for each region of interest (ROI) within lymphoid follicular regions (LFRs) of HPV-positive cases using the single-sample gene set enrichment analysis (ssGSEA) algorithm in GenePattern. Immune cell proportions for eosinophils and activated mast cells were estimated using CIBERSORTx. Correlations between ESs and immune cell proportions were assessed using Spearman’s rank correlation test, with statistical significance defined as *P* < 0.01. **(A, B)** ESs for early estrogen response were significantly positively correlated with eosinophil **(A)** and activated mast cell **(B)** proportions. **(C, D)** ESs for late estrogen response showed no significant correlation with eosinophil **(C)** or activated mast cell **(D)** proportions. (E) Early and late estrogen response ESs were strongly positively correlated. **(F)** Eosinophil and activated mast cell proportions were significantly positively correlated.

Notably, early estrogen response scores positively correlated with eosinophil and activated mast cell fractions (**Figures 3A, B**), whereas late estrogen response scores showed weaker associations (**Figures 3C, D**). Early and late estrogen responses were positively correlated with each other (**Figure 3E**). Eosinophil and mast cell fractions were intercorrelated (**Figure 3F**), suggesting coordinated Th2–hormonal modulation within the peritumoral lymphoid microenvironment.

### Histological validation

Immunohistochemistry confirmed spatially distinct eosinophil and mast cell infiltration patterns.

**Figure 4:**
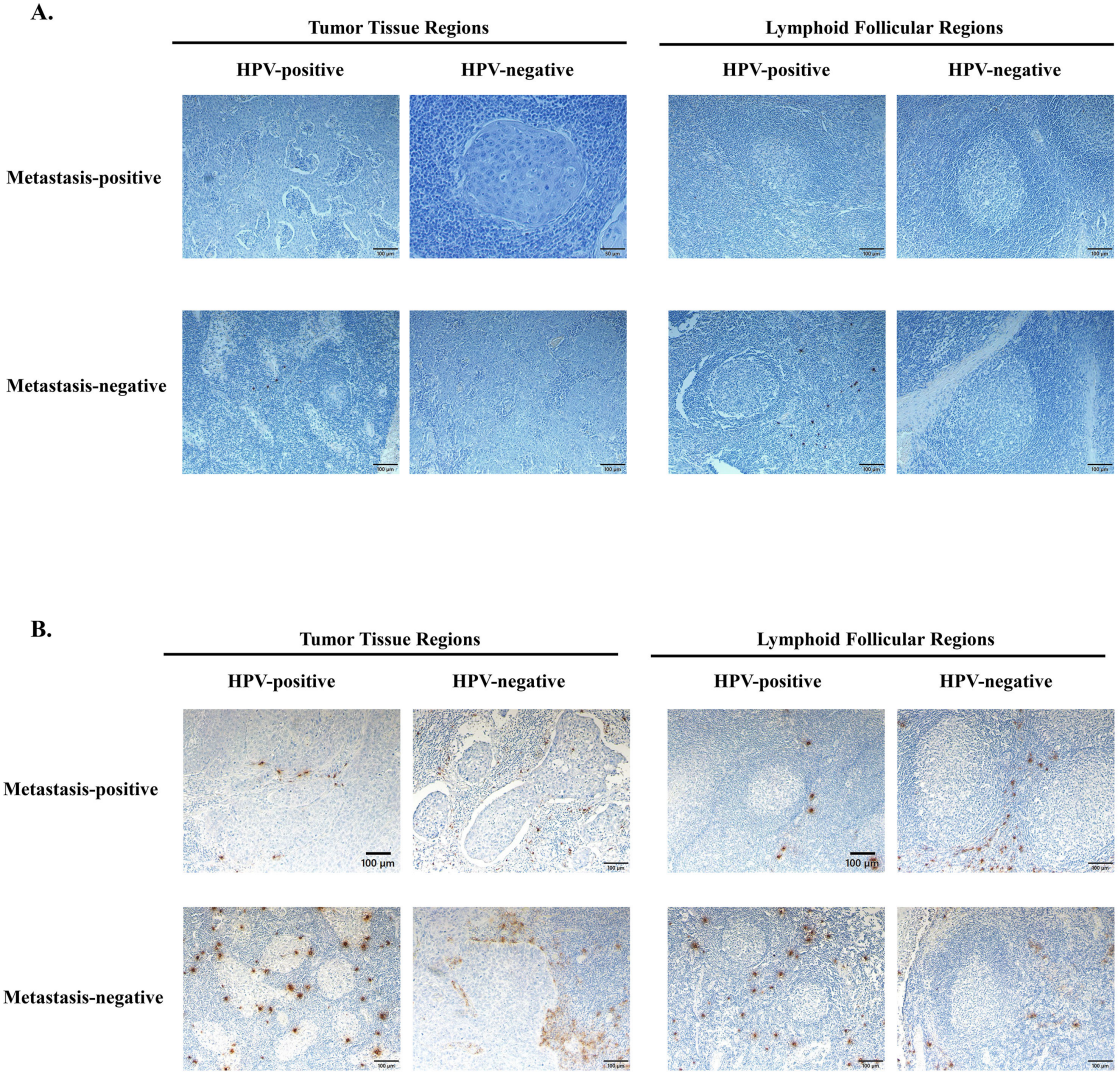
Immunohistochemical analysis of eosinophil and mast cell infiltration in oropharyngeal squamous cell carcinoma (OPC). **(A)** Representative immunohistochemical images of eosinophils detected by eosinophil major basic protein (EMBP) staining in formalin-fixed, paraffin-embedded OPC tissue sections. In HPV-positive, metastasis-negative tumors, eosinophils were distributed within both tumor tissue regions (TTRs) and adjacent lymphoid follicular regions (LFRs). In contrast, HPV-positive, metastasis-positive tumors exhibited minimal or no eosinophilic infiltration in either compartment. HPV-negative tumors, regardless of metastatic status, demonstrated only sparse eosinophil presence. Staining was visualized using diaminobenzidine (DAB) chromogen with methyl green counterstaining. **(B)** Representative immunohistochemical images of mast cells detected by tryptase staining in formalin-fixed, paraffin-embedded OPC tissue sections. In HPV-positive, metastasis-negative tumors, mast cells were observed in both TTRs and LFRs, whereas HPV-positive, metastasis-positive tumors exhibited reduced mast cell infiltration. HPV-negative tumors showed a comparable distribution of mast cells regardless of metastatic status. Staining was visualized using DAB chromogen with methyl green counterstaining. Scale bars: 100 μm.

EMBP staining revealed accumulation of eosinophils in TTRs and LFRs of non-metastatic HPV-positive tumors but not in metastatic or HPV-negative cases (**Figure 4A**). Mast cell tryptase staining showed an apparent increase in mast-cell density in HPV-positive non-metastatic tumors, although this observation was qualitative and not quantitatively assessed (**Figure 4B**).

These observations are consistent with transcriptomic data, underscoring the involvement of eosinophils and mast cells in the Th2-polarized immune microenvironment that may restrain lymphatic metastasis in HPV-positive OPCs.

## Discussion

This exploratory pilot study applied spatial transcriptomics and immune deconvolution to characterize the tumor–immune microenvironment of HPV-associated OPC. Despite the limited sample size, this analysis revealed distinct immune architectures, highlighting potential roles of B cell survival, Th2 polarization, and estrogen-related immune modulation in restricting lymphatic metastasis. While our previous work investigated peritumoral immune signatures using bulk transcriptomics (10, 22), the current study adds spatial context by resolving lymphoid microarchitecture and linking transcriptional profiles to immune cell composition and protein-level validation. To our knowledge, this represents the first spatial transcriptomic characterization of anatomically defined lymphoid compartments in HPV-related OPC performed using the GeoMx platform.

Clinically, HPV-positive OPC paradoxically presents as small primary tumors with frequent early cervical metastasis (23). The spatially resolved analysis demonstrated that LFRs adjacent to HPV-positive tumors exhibited transcriptional enrichment of anti-apoptotic B cell signaling, suggesting prolonged B cell survival and potentially sustained antigen presentation. Immune deconvolution supported this observation, showing enrichment of naïve and plasma B cells, M2 macrophages, eosinophils, and activated mast cells in non-metastatic tumors—features compatible with a Th2-skewed immune niche. Th2-associated immunity, while classically linked to tissue repair and antibody production, may in this context promote immune homeostasis and barrier maintenance, potentially constraining early lymphatic spread (24–26).

The B cell–innate immune cell axis observed here aligns with prior studies showing HPV-specific antibodies within the tumor microenvironment (7). Such antibodies may contribute to anti-tumor defense through antibody-dependent cellular cytotoxicity or immune complex formation, although this remains speculative. The consistent presence of naïve B cells in both tumor and lymphoid compartments suggests an active but incompletely matured immune process. Whether these cells represent a reservoir for adaptive activation or an immature immune state will require further functional exploration (27).

Eosinophils and mast cells were also enriched in non-metastatic HPV-positive tumors, consistent with a Th2-type immune milieu. These cells are increasingly recognized as modulators of cancer immunity (28–30). Eosinophils can release cytotoxic mediators, act as non-classical antigen-presenting cells, and interact bidirectionally with mast cells (31). Their co-occurrence and correlation in our dataset suggest coordinated recruitment, though causal relationships remain to be clarified. These findings hint that eosinophil–mast cell interactions may help sustain a localized, protective immune niche. Immunohistochemical validation supported transcriptomic observations: eosinophils and mast cells accumulated primarily in non-metastatic HPV-positive cases, confirming cellular heterogeneity between metastatic states. In contrast, HPV-negative tumors exhibited sparse immune infiltration and lacked structured immune signatures observed in HPV-positive cases. Although only two cases were analyzed, this difference reinforces that HPV-negative and -positive OPCs represent distinct immunobiological entities.

By contrast, metastatic HPV-positive tumors displayed enhanced T cell activation and inflammatory signaling, together with enrichment of bile acid metabolism pathways. While T cell activation is typically favorable, excessive or dysregulated responses may promote immune exhaustion or escape (32). The involvement of bile acid–related pathways is intriguing, as bile acids can regulate epithelial–mesenchymal transition and immune suppression in other cancers (33). Although noncanonical in OPC, such pathways may reflect microenvironmental or metabolic reprogramming that facilitates metastasis, meriting future investigation.

Of particular interest was the enrichment of estrogen response signatures in non-metastatic HPV-positive LFRs. Early estrogen response correlated with eosinophil and activated mast cell proportions, implying that local hormonal signaling might shape immune composition. Estrogen is known to modulate eosinophil activity, promote M2 macrophage differentiation, and enhance B cell maturation (28, 34). Estrogen receptor activation can also influence cytidine deaminase (AID/APOBEC) pathways with potential antiviral and immunoregulatory roles (35). Prior reports associate estrogen receptor expression with improved prognosis in HPV-positive OPC (36). Together, these observations suggest that local estrogen signaling could favor a Th2-biased immune microenvironment that limits metastasis, though this remains to be validated experimentally. From a translational perspective, these findings raise the hypothesis that reinforcing Th2-type immune activity or modulating estrogen signaling might strengthen local anti-tumor immunity. B cells, eosinophils, and mast cells could represent potential immunomodulatory targets, though current evidence remains preliminary.

## Limitations of the study

This study has several limitations inherent to its exploratory design. The cohort size was small, particularly for HPV-negative cases, precluding definitive conclusions and limiting reproducibility. Clinicopathologic factors such as age and stage were not adjusted between HPV groups, which may have introduced potential confounding effects. The sample size was primarily constrained by the limited availability of treatment-naïve FFPE specimens suitable for spatial transcriptomic analysis, as well as by the exploratory aim of generating preliminary spatial immune signatures. Only male patients were included, introducing possible sex-related bias, particularly relevant to estrogen-responsive signaling.

The limited sample size should be acknowledged as a key constraint that may reduce statistical power and generalizability. Nevertheless, the consistent spatial and immunological trends observed across cases support the exploratory value of our findings. Moreover, immune deconvolution using CIBERSORTx was exploratory; while useful for identifying compartmental trends, it does not fully capture spatial dependencies. The lack of OPC-specific single-cell reference data limited the use of spatially aware deconvolution tools such as SPOTlight or RCTD (37). Finally, immunohistochemical evaluation in this study was based on semi-quantitative visual assessment rather than objective digital quantification, which may reduce precision and reproducibility.

Future studies employing larger, sex-balanced cohorts with spatially resolved multi-omics and automated image analysis are warranted to validate and extend these observations.

This study used different statistical thresholds according to the analytical purpose: an exploratory cutoff of p < 0.05 for DEG identification and a more stringent p < 0.01 for validation analyses, ensuring both sensitivity and robustness of interpretation. The use of unadjusted p-values in the DEG analysis was justified by the small sample size and hypothesis-generating purpose of this study, as FDR correction would have eliminated potentially meaningful biological signals.

In summary, non-metastatic HPV-positive OPCs displayed a spatially organized, Th2-biased immune landscape enriched in B cells, eosinophils, and mast cells, accompanied by local estrogen signaling signatures. Conversely, metastatic tumors exhibited inflammatory and metabolic activation, including bile acid–related pathways. These contrasting immune architectures may influence the propensity for lymphatic metastasis and provide a foundation for future mechanistic and translational research. As spatial profiling technologies advance, integrating transcriptomic, proteomic, and hormonal analyses may yield deeper insights into HPV-associated tumor–immune interactions.

## Data Availability

The raw data supporting the conclusions of this article will be made available by the authors, without undue reservation.

## Supplementary material

Supplementary Figure 1 Principal component analysis (PCA) of normalized gene expression profiles from 2 lymphoid follicular regions (LFRs). (A) Comparison between HPV-positive and HPV-negative 3 oropharyngeal carcinoma (OPC) cases. The 95% confidence ellipses largely overlapped, and 4 PERMANOVA revealed no significant difference (F = 0.090, p = 0.100), indicating no evidence of 5 batch effects. (B) Comparison between metastasis-positive and metastasis-negative regions of interest 6 (ROIs) within HPV-positive OPC cases (n = 4). Only modest separation was observed, with a small 7 effect size despite statistical significance (F = 0.221, p = 0.028), suggesting that the trend is unlikely 8 to result from batch effects and is more consistent with biological variation. 9 10.

Supplementary Figure 2 Gene Ontology (GO) enrichment analysis of tumor tissue regions (TTRs) and 11 lymphoid follicular regions (LFRs) based on human papillomavirus (HPV) status. (A, C) 12 Volcano plots displaying differentially expressed genes (DEGs) between HPV-positive and HPV-13 negative samples in TTRs (A) and LFRs (C). Red and blue boxed regions indicate genes upregulated 14 in HPV-positive and HPV-negative samples, respectively. Statistical significance was defined as |log_2_| 15 fold change > 0.5 and –log_10_ P > 1.3. B and D: Proportions of significantly enriched GO terms in TTRs 16 (B) and LFRs (D) of HPV-negative samples compared with HPV-positive samples. The ClueGO plug-17 in for Cytoscape software version 3.10.3, was used to analyze and visualize functionally grouped GO 18 pathways associated with Biological Process, Cellular Component, Immune System Process, and 19 Molecular Function. A: Analysis of TTRs revealed 37 and 126 significantly upregulated genes in 20 HPV-positive and HPV-negative samples, respectively. B: In TTRs of HPV-negative samples, eight 21 significantly enriched GO terms were identified from the 126 upregulated genes compared with those 22 from HPV-positive samples. C: Analysis of LFRs revealed 31 and 193 significantly upregulated genes 23 in HPV-positive and HPV-negative samples, respectively. D: In LFRs of HPV-negative samples, 19 24 significantly enriched GO terms were identified from the 193 upregulated genes compared with those 25 from HPV-positive samples. 26 27.

Supplementary Figure 3 Gene Ontology (GO) enrichment analysis of tumor tissue regions (TTRs) and 28 lymphoid follicular regions (LFRs) in human papillomavirus (HPV)-positive oropharyngeal 29 carcinoma (OPC) based on lymph node metastasis status. 30 (A, D) Volcano plots displaying differentially expressed genes (DEGs) in TTRs (A) and LFRs (D) 31 between metastasis-positive and metastasis-negative HPV-positive OPC samples. Red and blue boxed 32 regions indicate genes upregulated in the metastasis-positive and metastasis-negative groups, 33 respectively. Statistical significance was defined as |log_2_| fold change > 0.5 and –log_10_ P > 1.3. The 34 ClueGO plug-in for Cytoscape software version 3.10.3, was used to analyze and visualize functionally 35 grouped GO pathways associated with Biological Process, Cellular Component, Immune System 36.

Supplementary Figure 4 Immune cell composition in HPV-positive oropharyngeal carcinoma based on lymph node metastasis status. Relative immune cell proportions were estimated using CIBERSORTx and compared between metastasis-negative and metastasis-positive samples. Statistical significance was assessed using the Mann–Whitney U test (P < 0.01). **(A)** In tumor tissue regions (TTRs), metastasis-negative samples exhibited significantly higher proportions of Th2-associated and innate immune cells, including naïve B cells, M2 macrophages, monocytes, activated mast cells, and eosinophils, compared with metastasis-positive samples. **(B)** In lymphoid follicular regions (LFRs), plasma cells, naïve B cells, M2 macrophages, activated mast cells, eosinophils, and neutrophils were significantly more abundant in metastasis-negative samples. **(C)** In contrast, activated dendritic cells were significantly elevated in LFRs of metastasis-positive samples, suggesting increased Th1 immune activity in these cases.

Supplementary Figure 5 Enrichment scores (ESs) from single-sample gene set enrichment analysis (ssGSEA) in lymphoid follicular regions (LFRs) of HPV-positive oropharyngeal carcinoma according to lymph node metastasis status. The association between nodal status and ESs from ssGSEA was evaluated in LFRs of HPV-positive cases using hallmark gene sets. Statistical analysis was performed using the Mann–Whitney U test, with significance set at P < 0.01. **(A)** In metastasis-positive LFRs, ESs for inflammation-related gene sets—HALLMARK_IL2_STAT5_SIGNALING, HALLMARK_INFLAMMATORY_RESPONSE, HALLMARK_INTERFERON_ALPHA_RESPONSE, and HALLMARK_INTERFERON_GAMMA_RESPONSE—were significantly elevated compared with metastasis-negative samples, indicating an upregulated inflammatory state. **(B)** ESs for HALLMARK_BILE_ACID_METABOLISM were significantly higher in metastasis-positive LFRs than in metastasis-negative ones, suggesting a role for bile acid signaling in lymphatic dissemination. **(C)** In contrast, ESs for HALLMARK_ESTROGEN_RESPONSE_EARLY and HALLMARK_ESTROGEN_RESPONSE_LATE were significantly increased in metastasis-negative LFRs, implicating estrogen signaling in shaping a local immune microenvironment associated with limited metastatic spread.
