# Supplementary figures and images for: Exploratory spatial transcriptomic profiling of peritumoral Th2 immune polarization in HPV-positive oropharyngeal cancer
Source: Front Oncol. 2025 Nov 26;15:1732480. doi: 10.3389/fonc.2025.1732480 (PMC12689367; doi:10.3389/fonc.2025.1732480)

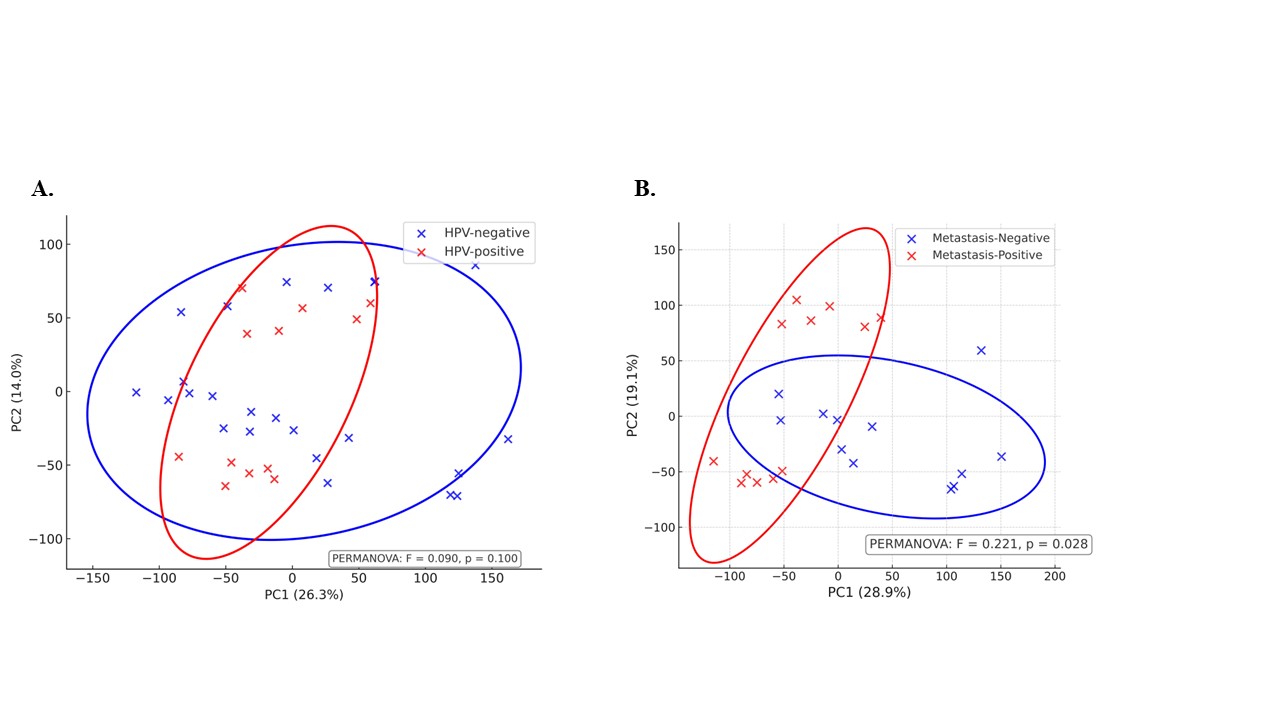

Supplement: Supplementary Figure 1 — Principal component analysis (PCA) of normalized gene expression profiles from 2 lymphoid follicular regions (LFRs). (A) Comparison between HPV-positive and HPV-negative 3 oropharyngeal carcinoma (OPC) cases. The 95% confidence ellipses largely overlapped, and 4 PERMANOVA revealed no significant difference (F = 0.090, p = 0.100), indicating no evidence of 5 batch effects. (B) Comparison between metastasis-positive and metastasis-negative regions of interest 6 (ROIs) within HPV-positive OPC cases (n = 4). Only modest separation was observed, with a small 7 effect size despite statistical significance (F = 0.221, p = 0.028), suggesting that the trend is unlikely 8 to result from batch effects and is more consistent with biological variation. 9 10. [file Image1.jpeg]

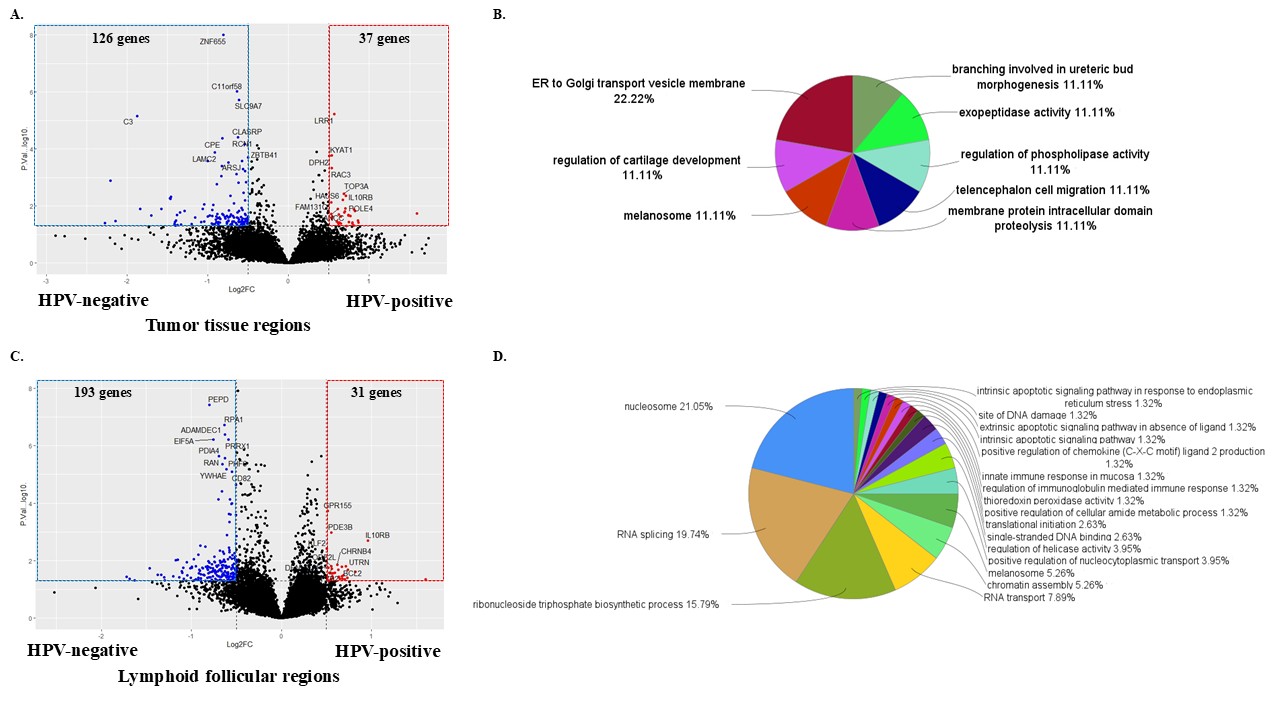

Supplement: Supplementary Figure 2 — Gene Ontology (GO) enrichment analysis of tumor tissue regions (TTRs) and 11 lymphoid follicular regions (LFRs) based on human papillomavirus (HPV) status. (A, C) 12 Volcano plots displaying differentially expressed genes (DEGs) between HPV-positive and HPV-13 negative samples in TTRs (A) and LFRs (C). Red and blue boxed regions indicate genes upregulated 14 in HPV-positive and HPV-negative samples, respectively. Statistical significance was defined as |log2| 15 fold change > 0.5 and –log10 P > 1.3. B and D: Proportions of significantly enriched GO terms in TTRs 16 (B) and LFRs (D) of HPV-negative samples compared with HPV-positive samples. The ClueGO plug-17 in for Cytoscape software version 3.10.3, was used to analyze and visualize functionally grouped GO 18 pathways associated with Biological Process, Cellular Component, Immune System Process, and 19 Molecular Function. A: Analysis of TTRs revealed 37 and 126 significantly upregulated genes in 20 HPV-positive and HPV-negative samples, respectively. B: In TTRs of HPV-negative samples, eight 21 significantly enriched GO terms were identified from the 126 upregulated genes compared with those 22 from HPV-positive samples. C: Analysis of LFRs revealed 31 and 193 significantly upregulated genes 23 in HPV-positive and HPV-negative samples, respectively. D: In LFRs of HPV-negative samples, 19 24 significantly enriched GO terms were identified from the 193 upregulated genes compared with those 25 from HPV-positive samples. 26 27. [file Image2.jpeg]

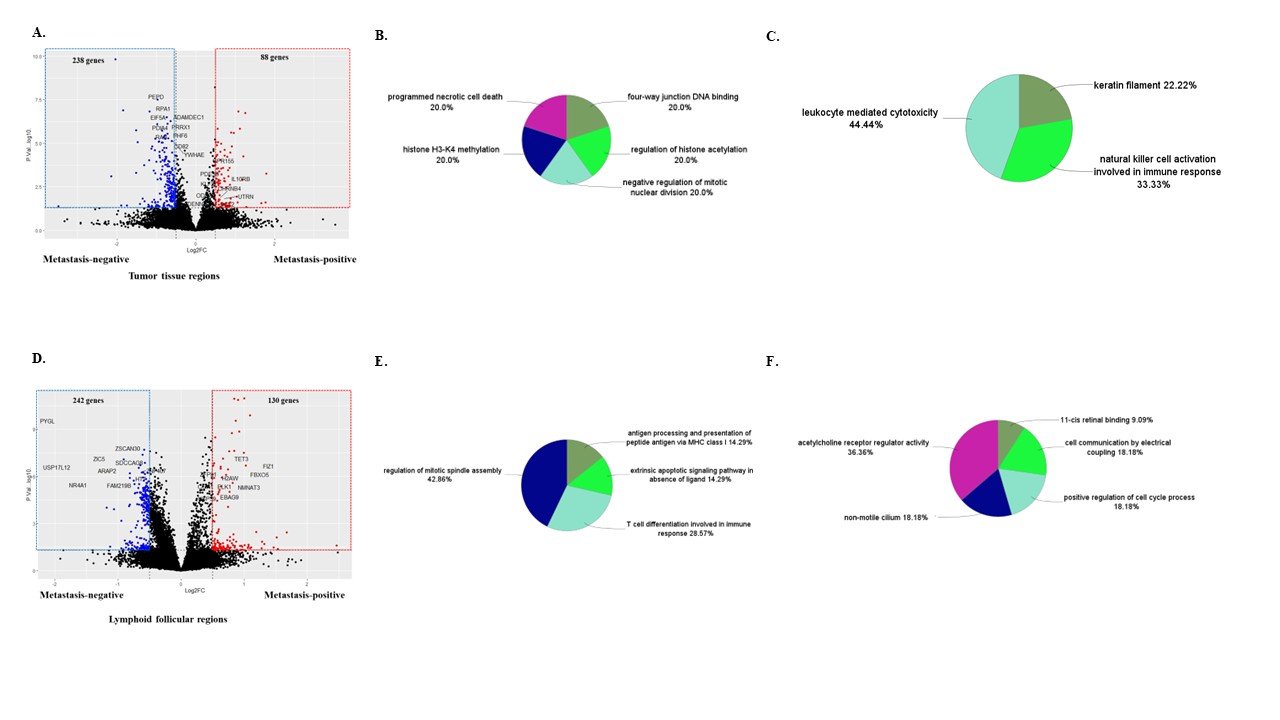

Supplement: Supplementary Figure 3 — Gene Ontology (GO) enrichment analysis of tumor tissue regions (TTRs) and 28 lymphoid follicular regions (LFRs) in human papillomavirus (HPV)-positive oropharyngeal 29 carcinoma (OPC) based on lymph node metastasis status. 30 (A, D) Volcano plots displaying differentially expressed genes (DEGs) in TTRs (A) and LFRs (D) 31 between metastasis-positive and metastasis-negative HPV-positive OPC samples. Red and blue boxed 32 regions indicate genes upregulated in the metastasis-positive and metastasis-negative groups, 33 respectively. Statistical significance was defined as |log2| fold change > 0.5 and –log10 P > 1.3. The 34 ClueGO plug-in for Cytoscape software version 3.10.3, was used to analyze and visualize functionally 35 grouped GO pathways associated with Biological Process, Cellular Component, Immune System 36. [file Image3.jpeg]

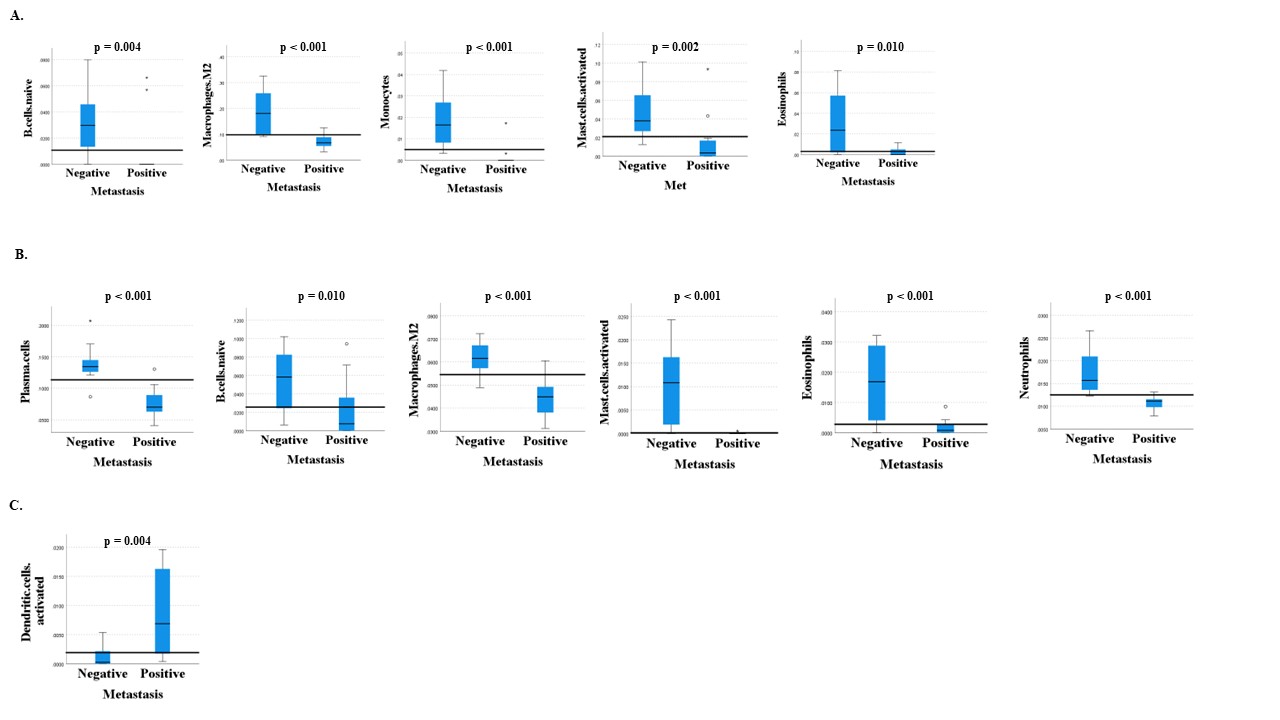

Supplement: Supplementary Figure 4 — Immune cell composition in HPV-positive oropharyngeal carcinoma based on lymph node metastasis status. Relative immune cell proportions were estimated using CIBERSORTx and compared between metastasis-negative and metastasis-positive samples. Statistical significance was assessed using the Mann–Whitney U test (P < 0.01). (A) In tumor tissue regions (TTRs), metastasis-negative samples exhibited significantly higher proportions of Th2-associated and innate immune cells, including naïve B cells, M2 macrophages, monocytes, activated mast cells, and eosinophils, compared with metastasis-positive samples. (B) In lymphoid follicular regions (LFRs), plasma cells, naïve B cells, M2 macrophages, activated mast cells, eosinophils, and neutrophils were significantly more abundant in metastasis-negative samples. (C) In contrast, activated dendritic cells were significantly elevated in LFRs of metastasis-positive samples, suggesting increased Th1 immune activity in these cases. [file Image4.jpeg]

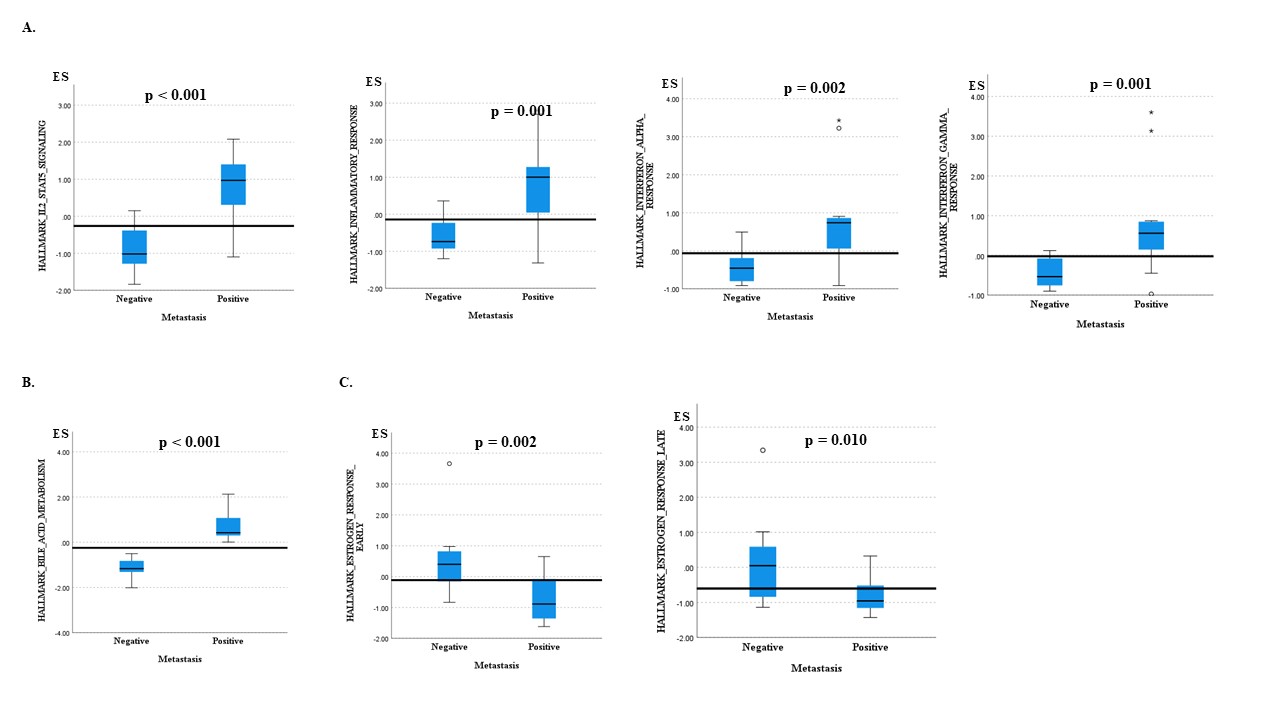

Supplement: Supplementary Figure 5 — Enrichment scores (ESs) from single-sample gene set enrichment analysis (ssGSEA) in lymphoid follicular regions (LFRs) of HPV-positive oropharyngeal carcinoma according to lymph node metastasis status. The association between nodal status and ESs from ssGSEA was evaluated in LFRs of HPV-positive cases using hallmark gene sets. Statistical analysis was performed using the Mann–Whitney U test, with significance set at P < 0.01. (A) In metastasis-positive LFRs, ESs for inflammation-related gene sets—HALLMARK_IL2_STAT5_SIGNALING, HALLMARK_INFLAMMATORY_RESPONSE, HALLMARK_INTERFERON_ALPHA_RESPONSE, and HALLMARK_INTERFERON_GAMMA_RESPONSE—were significantly elevated compared with metastasis-negative samples, indicating an upregulated inflammatory state. (B) ESs for HALLMARK_BILE_ACID_METABOLISM were significantly higher in metastasis-positive LFRs than in metastasis-negative ones, suggesting a role for bile acid signaling in lymphatic dissemination. (C) In contrast, ESs for HALLMARK_ESTROGEN_RESPONSE_EARLY and HALLMARK_ESTROGEN_RESPONSE_LATE were significantly increased in metastasis-negative LFRs, implicating estrogen signaling in shaping a local immune microenvironment associated with limited metastatic spread. [file Image5.jpeg]
